# Characteristics and health‐status outcomes in patients with atrial fibrillation detected via health screening

**DOI:** 10.1002/clc.23932

**Published:** 2022-10-27

**Authors:** Terumasa Yamashita, Takehiro Kimura, Nobuhiro Ikemura, Nozomi Niimi, Ippei Tsuzuki, Yuta Seki, Susumu Ibe, Kenji Hashimoto, Hiroshi Miyama, Taishi Fujisawa, Yoshinori Katsumata, Kojiro Tanimoto, Keiichi Nagami, Masahiro Suzuki, Shun Kohsaka, Keiichi Fukuda, Seiji Takatsuki

**Affiliations:** ^1^ Department of Cardiology Keio University School of Medicine Tokyo Japan; ^2^ Department of Cardiology Tokyo Medical Center Tokyo Japan; ^3^ Department of Cardiology Keiyu Hospital Tokyo Japan; ^4^ Department of Cardiology National Hospital Organization Saitama Hospital Tokyo Japan

**Keywords:** anticoagulation, atrial fibrillation, health screening, quality of life, symptom

## Abstract

**Background:**

Early detection of atrial fibrillation (AF) is important. Japan has a universal screening system, and regular health screening (HS) is available to support AF detection without a hospital visit. However, health‐related outcomes and other characteristics of HS‐detected and conventionally diagnosed AF remain unknown.

**Hypothesis:**

That the characteristics and health‐related outcomes of patients with HS‐detected AF may differ from those of patients whose AF was detected by other procedures.

**Methods:**

In total, 3318 consecutive newly referred AF cases were enrolled; demographic characteristics and health‐related and clinical outcomes were compared between two groups created based on the mode of AF detection (the HS and non‐HS groups). Health‐related outcomes were assessed using the AF Effect on QualiTy‐of‐life (AFEQT) questionnaire at baseline and after 1 year of follow‐up.

**Results:**

AF was detected by HS in 25.0% of patients; these patients had lower CHADS_2_ scores (1.01 vs. 1.50, *p* < .001), higher prevalence of persistent AF (odds ratio, 95% confidence interval; 2.21, 1.88–2.60) and asymptomatic presentation (3.19, 2.71–3.76), and better baseline QoL scores (83.6 vs. 75.0; *p* < .001). Catheter ablation was more frequently performed in the HS group at follow‐up (44.4% vs. 34.1%; *p* < .001). At 1‐year follow‐up, the AFEQT scores of the HS group were significantly better in most subdomains.

**Conclusions:**

In the Japanese registry, AF was detected via HS in 25% of patients referred to specialty centers for management. Notably, the overall health status of patients with HS‐detected AF improved after medical interventions, including catheter ablations.

## 1 INTRODUCTION

Atrial fibrillation (AF) has a substantial prevalence; its lifetime risk after 40 years of age is one‐in‐four.^1^ AF is associated with an increased risk of death, stroke, and impaired quality of life (QoL). Therefore, opportunistic AF screening is recommended for elderly patients and those with high stroke risk. In some cases, AF is asymptomatic or intermittent, making its diagnosis challenging. Data of patients with implanted cardiac devices indicated that asymptomatic AF represents up to a third of the total AF population. Furthermore, patients with subclinical AF have a higher stroke risk.^2^ However, these reports are based on studies that assessed patients who received some medical interventions. Japan introduced a universal screening system in the 1960s, and regular health screening (HS) programs are universally available to support AF detection without a hospital visit. The effectiveness of HS in the general population is unknown. This study aimed to assess differences in characteristics and health‐related outcomes between patients in whom AF was detected by HS and those in whom it was detected using other procedures.

## 2 METHODS

### 2.1 Data sources

The rationale and design of the Keio Interhospital Cardiovascular Studies for Atrial Fibrillation (KiCS‐AF) registry have been previously described.^3^ Briefly, the KiCS‐AF was a multicenter, registry‐based, retrospective cohort study of data on clinical variables and outcomes of consecutive newly diagnosed patients with AF diagnosis in an outpatient clinic at any one of 11 participating tertiary care hospitals within the Kanto area in Japan. To recruit treatment‐naive patients, the registry included only patients with AF who had been newly referred to network hospitals within the previous 6 months. Data on patient backgrounds, symptoms, use of medications such as oral anticoagulants (OACs), electrocardiogram (ECG), and blood sampling test results were collected from medical records.

Annual follow‐up examinations were conducted on all patients via mail, phone interviews, and medical chart reviews. Dedicated study coordinators updated the status of major cardiovascular events, laboratory test results, procedures performed, and subsequent changes in medications. Data quality assurance was achieved via quantitative reporting of data completeness (%), thorough quarterly education, and training of dedicated clinical research coordinators. Supervision by the senior study coordinator (I. U.) and exclusive on‐site auditing by study investigators (T. K., S. K., and S. T.) ensured proper registration of each patient. The institutional review board approved the protocol of all institutions, and the study was conducted in accordance with the Declaration of Helsinki. All participants provided written informed consent.

### 2.2 Assessments of the patients’ health status

In addition to the traditional data collected by healthcare providers, the KiCS‐AF collected patient‐reported outcomes using the internationally validated atrial fibrillation effect on quality‐of‐life (AFEQT; http://www.afeqt.org) questionnaire.^4^ Patients were requested to complete a questionnaire at registration and at the 1‐year follow‐up visit or by mail. The AFEQT is a 20‐item questionnaire that quantifies four domains of AF‐related QoL. These domains include symptoms, daily activities, treatment concerns, and treatment satisfaction, which were obtained using a seven‐point Likert response scale. The overall summary score is calculated from the first three domains, ranging from 0 (worst heath status) to 100 (best possible health status with no impairment). A five‐point change in the AFEQT Overall Summary score was observed among patients whose European Heart Rhythm Association functional status class changed by one; this change was clinically important.^5^

### 2.3 HS

The HS system in Japan was introduced in 1954. Since then, Japanese people have undergone HS once or twice a year. Risk factor, cancer, and cardiovascular disease screenings are conducted depending on the patient's choice. Mass screening is performed at schools, workplaces, and in the community by local government authorities. A systematic, whole‐body examination, called a human dry dock, is another type of HS popular among businesspeople. Typically, a routine check‐up involves staying at a clinic or hospital for several days to undergo thorough physical examinations. These two HS pathways are fundamental to the Japanese healthcare system. These programs usually include a 12‐lead ECG examination.

### 2.4 Study population

All data available until the 1‐year follow‐up examination of patients who were enrolled between September 12, 2012, and May 16, 2018, were included. At the time, 2742 (82.6%) of 3318 consecutive registered outpatients with AF had available 1‐year follow‐up data. At follow‐up, patients answered the AFEQT questionnaire at an average of 414 ± 72.0 (*n* = 2784) days after registration. There were 2092 patients in the non‐HS group and 692 in the HS group. We compared patient background characteristics, AFEQT scores, and clinical outcomes between the two groups. This cohort underwent follow‐up after 1 year for the occurrence of hospital admission, heart failure, stroke, bleeding, acute coronary syndrome, and death.

### 2.5 Statistical analyses

The data are presented as mean ± standard deviation (SD), percentage, or number of cases. Asymptomatic AF was defined as AF without any symptoms such as palpitations, dyspnea, fatigue, dizziness, chest pain, and syncope. Differences in characteristics and QoL assessments between the two groups were compared using the student's *t*‐test for continuous variables and *χ* ^2^ test for categorical variables. The paired *t*‐test was used to compare the AFEQT scores between baseline and follow‐up. Odds ratios (ORs) and 95% confidence intervals (95% CIs) were subsequently calculated. Statistical significance was set at *p* < .05. All statistical analyses were performed using SPSS version 26.0 (IBM Corp.).

## 3 RESULTS

### 3.1 Study population

The study population consisted of 3318 registered patients with AF (men, *n* = 2268; age, 68 ± 12 years; CHADS_2_ score, 1.4 ± 1.2; CHA_2_DS_2_‐VASc score, 2.5 ± 1.7). Persistent AF was identified in 1450 (44.4%) patients. Among them, 3291 (99.2%) and 2768 (83.4%) had ECG data recorded at baseline and 1‐year follow‐up visit, respectively. AF‐related symptoms were reported in 3318 (100%) patients.

### 3.2 Characteristics of patients with AF detected via HS

**Figure 1:**
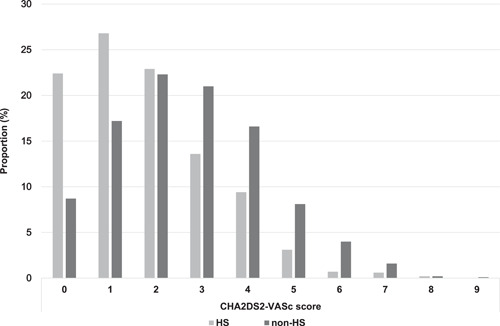
Proportion of CHA_2_DS_2_‐VASc score in the HS and non‐HS groups. The HS group (indicated by gray bars) has lower CHA_2_DS_2_‐VASc scores than the non‐HS group (indicated by black bars). HS, health screening.

**Figure 2:**
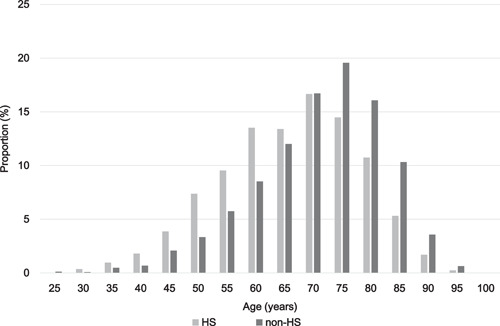
Histogram of age of patients in the HS and non‐HS groups. The distribution shows significantly younger patients in the HS group (indicated by gray bars) compared with the non‐HS group (indicated by black bars). HS, health screening.

A total of 829 (24.9%) patients had AF detected via HS. The baseline characteristics of the HS and non‐HS groups are summarized in Table 1. The HS group was more likely to be younger and less likely to be female; patients in this group had less comorbidities compared with the non‐HS group. Consequently, the HS group had lower CHADS_2_ and CHA_2_DS_2_‐VASc scores than the non‐HS group (Figure 1). A histogram of age in each group is shown in Figure 2. The proportion of patients aged 35–60 years was significantly higher and that of patients aged 70–95 years was significantly lower in the HS group than in the non‐HS group. The HS group was more likely to have persistent AF and a higher heart rate. Furthermore, serum brain natriuretic peptide levels were significantly lower in the HS group than in the non‐HS group. Regarding baseline treatments, the proportion of patients receiving either anticoagulants or antiplatelet agents was significantly higher in the non‐HS group. The proportion of patients receiving anticoagulants was significantly higher in the HS group at 1‐year follow‐up.

**Table 1:** Symptoms, comorbidities, and background characteristics of patients with atrial fibrillation detected via health screening

	All, *n* = 3318 (%)	HS, *n* = 829 (%)	non‐HS, *n* = 2489 (%)	*p* value	OR	95% CI
Background						
Age	67.8 ± 11.6	64.1 ± 12.0	69.1 ± 11.2	<.001	N/A	N/A
Height (cm)	164.2 ± 9.6	166.8 ± 9.1	163.4 ± 9.6	<.001	N/A	N/A
Weight (kg)	64.1 ± 13.4	67.5 ± 12.8	63 ± 13.4	<.001	N/A	N/A
Body mass index	23.6 ± 3.7	24.2 ± 3.5	23.5 ± 3.7	<.001	N/A	N/A
Heart failure	544 (16.4)	65 (7.8)	479 (19.2)	<.001	0.357	0.272–0.469
Hypertension	1911 (57.6)	402 (48.5)	1509 (60.7)	<.001	0.611	0.521–0.715
Age >75 years	1029 (31.0)	170 (20.5)	859 (34.5)	<0.001	0.49	0.406–0.591
Age >65 years	2181 (65.7)	430 (51.9)	1751 (70.3)	<0.001	0.454	0.387–0.534
Diabetes	546 (16.5)	122 (14.7)	424 (17)	.121	0.841	0.676–1.047
Stroke	278 (8.4)	42 (5.1)	236 (9.5)	<.001	0.509	0.363–0.714
Vascular disease	381 (11.5)	29 (3.5)	352 (14.2)	<.001	0.22	0.149–0.324
CAD	308 (9.3)	17 (2.1)	291 (11.7)	<.001	0.158	0.096–0.26
Female	1050 (31.6)	174 (21)	876 (35.2)	<.001	0.489	0.406–0.589
CHADS/CHADS‐VASc						
CHADS_2_	1.4 ± 1.2	1 ± 1.1	1.5 ± 1.2	<.001	N/A	N/A
CHA_2_DS_2_‐VASc	2.5 ± 1.7	1.8 ± 1.5	2.7 ± 1.7	<.001	N/A	N/A
ECG						
Persistent atrial fibrillation	1450 (44.4)	479 (59.1)	971 (39.6)	<.001	2.209	1.879–2.597
Sinus rhythm	1568 (47.6)	278 (34.0)	1290 (52.2)	<.001	2.118	1.796–2.498
Heart rate (beats/min)	78.7 ± 17.7	80.1 ± 16.9	78.2 ± 18	.009	N/A	N/A
Echocardiography						
LVEF (%)	57.5 ± 8.2	58.3 ± 6.7	57.2 ± 8.7	.001	N/A	N/A
LA (cm)	4.1 ± 0.8	4.2 ± 0.7	4.1 ± 0.8	.024	N/A	N/A
Laboratory						
Cr (mg/dl)	1 ± 0.7	0.9 ± 0.6	1 ± 0.7	.116	N/A	N/A
Ccr (mL/min)	71.7 ± 28.4	79.7 ± 27.2	69.1 ± 28.3	<.001	N/A	N/A
AST (IU/L)	26.5 ± 15.3	26.7 ± 18	26.4 ± 14.4	.633	N/A	N/A
PT‐INR	1.3 ± 0.5	1.3 ± 0.4	1.3 ± 0.5	.004	N/A	N/A
APTT (s)	36.5 ± 10.7	35.3 ± 9.1	36.9 ± 11.2	.003	N/A	N/A
BNP (pg/ml)	148.4 ± 189.3	121.8 ± 118.6	157.8 ± 208	<.001	N/A	N/A
Symptoms						
Palpitations	1398 (42.1)	220 (26.5)	1178 (47.3)	<.001	0.402	0.338–0.478
Dyspnea	563 (17.0)	92 (11.1)	471 (18.9)	<0.001	0.535	0.421–0.679
Dizziness	134 (4.0)	21 (2.5)	113 (4.5)	.011	0.546	0.341–0.877
Fatigue	129 (3.9)	26 (3.1)	103 (4.1)	.196	0.75	0.484–1.162
Chest pain	91 (2.7)	11 (1.3)	80 (3.2)	.004	0.405	0.215–0.764
Syncope	54 (1.6)	5 (0.6)	49 (2.0)	.007	0.302	0.12–0.761
Asymptomatic	1356 (40.9)	514 (62)	842 (33.8)	<.001	3.192	2.712–3.757
Previous histories						
Dyslipidemia	1201 (36.2)	246 (29.7)	955 (38.4)	<.001	0.677	0.572–0.802
COPD	81 (2.4)	10 (1.2)	71 (2.9)	.008	0.415	0.213–0.809
Thyroid dysfunction	76 (2.3)	14 (1.7)	62 (2.5)	.182	0.673	0.375–1.208
Hemodialysis	24 (0.7)	2 (0.2)	22 (0.9)	.059	0.271	0.064–1.155
Malignant disorder	87 (2.6)	12 (1.4)	75 (3.0)	.014	0.472	0.256–0.873
Smoking	544 (16.4)	152 (18.4)	392 (15.8)	.081	1.202	0.978–1.477
Therapy						
Anticoagulation	2773 (83.6)	661 (79.8)	2112 (84.9)	.001	0.705	0.576–0.862
Warfarin	432 (13.0)	92 (11.1)	340 (13.7)	.059	0.79	0.618–1.009
DOACs	2342 (70.6)	570 (68.8)	1772 (71.2)	.183	0.89	0.751–1.056
Antiplatelets	416 (12.5)	47 (5.7)	369 (14.8)	<.001	0.346	0.252–0.473
Aspirin	311 (9.4)	35 (4.2)	276 (11.1)	<.001	0.354	0.247–0.507
Clopidogrel	110 (3.3)	9 (1.1)	101 (4.1)	<.001	0.26	0.131–0.516
ACE	1198 (36.1)	244 (29.5)	954 (38.4)	<.001	0.671	0.566–0.796
Beta blocker	1775 (53.5)	376 (45.4)	1399 (56.2)	<.001	0.648	0.553–0.758
Ca blocker	1330 (40.1)	271 (32.7)	1059 (42.6)	<.001	0.657	0.556–0.775
Digitalis	203 (6.1)	32 (3.9)	171 (6.9)	.002	0.544	0.37–0.801
Diuretics	714 (21.5)	97 (11.7)	617 (24.8)	<.001	0.402	0.32–0.507
Rhythm control	1768 (53.4)	467 (56.3)	1301 (52.4)	.049	1.172	1.001–1.373
Previous ablation	216 (6.5)	38 (4.6)	178 (7.2)	.010	0.625	0.436–0.895

*Note*: Values reflect mean ± standard deviation or *n* (%).

Abbreviations: ACE, angiotensin‐converting enzyme; APTT, activated partial thromboplastin time; AST, aspartate transaminase; BNP, brain natriuretic peptide; CAD, coronary artery disease; Ccr, creatinine clearance; CHA2DS2‐VASc (cardiac failure; hypertension; age ≥75 years [doubled]; diabetes; previous stroke or TIA [doubled]; vascular disease; age 65–74 years; and sex category); CHADS2 (cardiac failure; hypertension; age ≥ 75 years; diabetes; previous stroke or transient ischemic attack [TIA] [doubled]); CI, confidence interval; COPD, chronic obstructive pulmonary disease; Cr, creatinine; DOAC, direct oral anticoagulant; ECG, electrocardiogram; HS, health screening; LA, left atrium; LVEF, left ventricular ejection fraction; N/A, not available; OR, odds ratio; PT‐INR, prothrombin time–international normalized ratio.

### 3.3 Comparison of outcomes

A comparison of outcomes at 1‐year follow‐up is presented in Supporting Information: Table 1. The proportion of patients with sinus rhythm was significantly lower in the HS group than in the non‐HS group at baseline (Table 1); this did not differ between the two groups at the 1‐year follow‐up visit (Supporting Information: Table 1). Hence, the increase in the proportion of patients with sinus rhythm was more significant in the HS group than in the non‐HS group (34.3% vs. 17.0%; *p* < .001). The incidence of admission (due to heart failure) and death was significantly higher in the non‐HS group, whereas the incidence of stroke, bleeding, and acute coronary syndrome did not differ between the two groups.

### 3.4 Assessment of QoL

**Figure 3:**
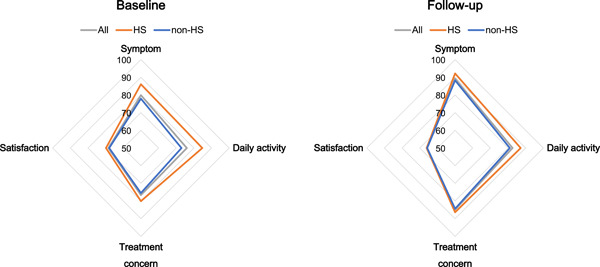
Differences in QoL scores between the HS and non‐HS groups both at baseline and follow‐up. The HS group has significantly higher QoL scores (except for the satisfaction domain) than the non‐HS group at baseline and follow‐up. The vertices of the square indicate each domain (symptom, daily activity, treatment, and satisfaction). Gray, red, and blue lines indicate all patients, HS group, and non‐HS group, respectively. HS, health screening; QoL, quality of life.

The QoL scores of 3299 (99.4%) and 2742 (82.6%) patients were obtained at baseline and after 1 year of follow‐up, respectively. The proportion of patients with asymptomatic AF was doubled while complaints of palpitations were reduced by almost half in the HS group compared with the non‐HS group. A comparison of QoL scores between the non‐HS and HS groups both at baseline and follow‐up is shown in Figure 3.

**Figure 4:**
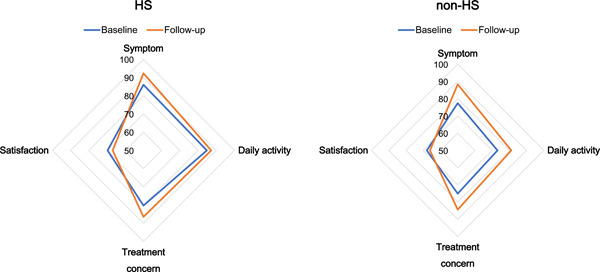
Difference in QoL scores between the baseline and follow‐up data in both the HS and non‐HS groups. QoL scores (except for satisfaction) increased after 1 year of follow‐up in both the HS and non‐HS groups. The vertices of the square indicate each domain (symptom, daily activity, treatment, and satisfaction). Red and blue lines indicate the HS group and non‐HS group, respectively. HS, health screening; QoL, quality of life.

The HS group had significantly higher baseline AFEQT scores for all domains (symptom, daily activity, treatment concern, and satisfaction). At the 1‐year follow‐up visit, the AFEQT scores were significantly better in all domains except for the satisfaction domain in the two groups. A detailed comparison of each QoL questionnaire is provided in Supporting Information: Table 2. The answers to almost all questions were significantly better in the HS group, while concerns about the side effects of medications and blood thinners became insignificant after follow‐up. Comparisons of QoL scores between baseline and follow‐up data of both the HS and non‐HS groups are shown in Figure 4. In the non‐HS group, the improvement in the QoL was statistically significant in all domains except for the satisfaction domain. Although the baseline scores were better in the HS group, improvement in the symptom and treatment concerns was statistically significant in them. A detailed comparison of each question is provided in Supporting Information: Table 3. The score for the question “feeling worried that AF can start at any time” was only improved in the non‐HS group. Although the proportion of asymptomatic patients was significantly higher in the HS group, the scores for the questions regarding activities such as “doing things with friends” and “walking briskly” were significantly higher in the HS group. In contrast, they largely remained unchanged in the non‐HS group.

## 4 DISCUSSION

In this multicenter Japanese registry, approximately one‐quarter of patients had their AF detected via HS. HS‐detected AF was characterized as asymptomatic, persistent, and more prevalent in the young population. More than half of patients in the HS group were asymptomatic, which might be because symptomatic patients would visit the hospital for diagnosis. More persistent AF was included in the HS group since paroxysmal AF was associated with a more symptomatic presentation. In addition, a single ECG examination in HS hardly detected infrequent paroxysmal AF. In the present study, patients in the HS group were younger than those in the non‐HS group, while patients with persistent AF were expected to be older than those with paroxysmal AF.^6^

We showed that medical interventions could improve the outcomes of patients in the HS group with relatively higher baseline QoL scores. In the present study, a considerable number of patients in the HS group started anticoagulation therapy and underwent catheter ablation. Consequently, the HS group had a higher rate of increase in sinus rhythm at the 1‐year follow‐up examination. Furthermore, among patients in the HS group who did not receive anticoagulants at baseline, concerns about the side effects of blood thinners did not significantly change between baseline and the 1‐year follow‐up visit (Supporting Information: Table 4), even though 49% of them were started on anticoagulants. This finding contributes to the improved QoL observed in the HS group. Therefore, HS may be useful in detecting patients with AF that are eligible for catheter ablation. Our findings highlight the significance of HS in proactive and opportunistic AF detection and demonstrate that a considerable number of patients with permanent AF remain asymptomatic among the younger age group. Furthermore, relying on data from this cohort, patients’ subjective complaints may have a low diagnostic value in AF detection. In a previous report, repeated annual ECG screening detected new AF cases each year. Most HS‐detected AF cases had a class‐1 anticoagulation recommendation in the population over 65 years old.^7^ Although the cost‐effectiveness of screening the whole population, including young people, is controversial, the improvement in QoL with medical intervention is obvious. Therefore, HS‐detected AF should be followed by medical care.

### 4.1 Comparison with previous reports

Disease screening for cancer is standard in the United States and in European countries. Nevertheless, health check‐ups are limited. Therefore, reports on HS‐detected AF are lacking. However, those who consulted for a health check‐up reportedly have lower cardiovascular risk factors.^8^

The 12‐lead ECG test is included in the Japanese HS. While this test is critical for AF detection, only a few countries or medical systems have it as part of their screening programs. In Turkey, the REALISE AF trial enrolled patients on a single‐visit basis and performed ECGs; they reported that AF‐related symptoms were evident in 89.2% of the population.^9^ This rate is much higher than the prevalence of symptoms in our registry (58.8%), reflecting the significance of HS in AF detection. Clinically, AF with a low CHADS_2_ score, as seen in the young population, may not correspond to a high risk of thromboembolism. However, asymptomatic patients remain undetected unless HS is universally employed. HS may reduce the adverse effects of AF.

### 4.2 Stroke prevention in HS‐detected AF

More than 80% of the selected patients in our registry received OACs, which was considerably higher than the numbers reported in previous AF studies.^10^ The current guidelines for anticoagulation prefer the use of the CHA_2_DS_2_‐VASc score to the CHADS_2_ score.^1^ Borderline patients (e.g., those with a CHADS_2_ score of 1 or a CHA_2_DS_2_‐VASc score of 1–2) are considered ideal candidates for direct OACs since they (particularly East Asians) have a better safety profile.^11^ In the current study, direct OACs were prescribed six times more frequently than warfarin, suggesting that direct OACs were preferred in Japan. This may be important in implementing HS for AF detection since patients’ stroke risk in the HS group was lower than that in conventional patients with AF.

### 4.3 HS‐detected AF and QoL

Promoting an aggressive therapeutic strategy might be difficult in a population with a well‐preserved QoL. The RACE II trial showed that the stringency of heart rate control did not influence QoL.^12^ However, the parameters that affect QoL are not fully understood. The FRACTAL study showed that sex, age, and comorbid conditions were strongly associated with QoL in new‐onset AF.^13^ Our registry revealed that the QoL in all domains was better in the HS group. Moreover, the J‐RHYTHM II study showed that the frequency of asymptomatic episodes was correlated with QoL reduction^14^ due to AF attacks during sleep and cardiac output. In contrast, the type of AF and rhythm were irrelevant to QoL. A substudy of the STAR AF trial showed that QoL was generally improved regardless of the outcome,^15^ suggesting that a placebo effect might alter QoL.^16^ The time from the initial diagnosis to registration might also affect the QoL scores.^17^ Thus, the interpretation of QoL results was often limited in understanding meaningful changes. The underlying parameters affecting QoL should be further evaluated to construct a rationale for applying adequate therapy globally, even for patients with preserved QoL and low risk.

### 4.4 Limitations

The present study was conducted in a single medical network within the metropolitan Tokyo area. Thus, the results might not be reproducible in all populations. In other words, the generalizability of our findings to individuals of different ages, races, or ethnicities is uncertain. Additionally, this registration enrolled many patients referred for catheter ablation. Therefore, patients’ background might not be identical to that of the general Japanese population. Furthermore, the benefit of OACs in patients with AF was typically studied in symptomatic patients with standard ECG diagnosis, while the advantage in patients diagnosed through mass screening programs remains unclear. Moreover, HS may not be suitable for detecting asymptomatic paroxysmal AF in young patients with few comorbidities; this is a challenge that needs to be addressed in the future. Although the government mandates HS, employers may refuse to undergo ECG examinations or other screening programs. Hence, both methods of data collection have limitations.

## 5 CONCLUSIONS

In the Japanese registry, AF was detected via HF in 25% of the patients referred to specialized centers for treatment. Notably, the overall health of patients with HS‐detected AF improved following medical interventions such as catheter ablations.

## CONFLICT OF INTEREST

This study was funded by Bayer Yakuhin Ltd.

## Supporting information

Supporting information.

## Data Availability

The deidentified participant data will not be shared.
