# Supplementary material for: Characteristics and health‐status outcomes in patients with atrial fibrillation detected via health screening
Source: Clin Cardiol. 2022 Oct 27;46(1):32–40. doi: 10.1002/clc.23932 (PMC9849435; doi:10.1002/clc.23932)
Supplement: Supplementary file 1 — Supporting information. [file CLC-46-32-s001.docx]

**Supplementary Table 1.** Outcomes and status of patients with atrial fibrillation detected via health screening one year after registration

|  | All n = 3318 (%) | HS n = 829 (%) | non-HS n = 2489 (%) | p value | OR | 95% CI |
| --- | --- | --- | --- | --- | --- | --- |
| Follow-up ECG data collected | 2220 (80.2) | 537 (79.8) | 1683 (80.3) | 0.759 | 0.967 | 0.778–1.201 |
| Sinus rhythm | 1516 (68.2) | 368 (68.5) | 1148 (68.2) | 0.891 | 0.985 | 0.800–1.215 |
| Rate of increase in sinus rhythm | 467 (21.2) | 183 (34.3) | 284 (17.0) | <0.001 | 2.555 | 2.052–3.183 |
| Antiplatelet | 284 (10.4) | 33 (4.9) | 251 (12.2) | <0.001 | 0.37 | 0.254–0.537 |
| Discontinuation of antiplatelets | 114 (32.9) | 15 (44.1) | 99 (31.7) | 0.145 | 1.699 | 0.829–3.482 |
| Anticoagulation | 1772 (64.8) | 441 (65.3) | 1331 (64.4) | 0.744 | 1.031 | 0.859–1.237 |
| Discontinuation of anticoagulants | 696 (30.2) | 169 (31.0) | 527 (29.9) | 0.648 | 1.05 | 0.852–1.293 |
| Start of anticoagulants | 161 (37.7) | 64 (49.6) | 97 (32.6) | 0.001 | 2.040 | 1.340–3.107 |
| Antiarrhythmic drugs | 386 (14.2) | 85 (12.6) | 301 (14.7) | 0.185 | 0.84 | 0.649–1.087 |
| Hospital admission | 1598 (51.9) | 418 (54.9) | 1180 (50.9) | 0.057 | 1.173 | 0.995–1.383 |
| Catheter ablation | 1128 (36.7) | 338 (44.4) | 790 (34.1) | <0.001 | 1.543 | 1.306–1.824 |
| Heart failure | 79 (2.6) | 9 (1.2) | 70 (3.0) | 0.005 | 0.384 | 0.191–0.772 |
| Stroke | 22 (0.7) | 4 (0.5) | 18 (0.8) | 0.475 | 0.675 | 0.228–1.999 |
| Bleeding | 45 (1.5) | 13 (1.7) | 32 (1.4) | 0.515 | 1.24 | 0.648–2.376 |
| ACS | 4 (0.1) | 1 (0.0) | 3 (0.1) | 1.000 | 1.015 | 0.105–9.776 |
| Death | 41 (1.3) | 4 (0.5) | 37 (1.6) | 0.025 | 0.325 | 0.116–0.916 |

Values reflect n (%).

ACS, acute coronary syndrome; CAD, coronary artery disease; CI, confidence interval; ECG, electrocardiogram; HS, health screening; OR, odds ratio

**Supplementary Table 2.** Comparison of the QoL score in the HS and non-HS groups

**QoL score at baseline**

|  | All n = 3318 | HS n = 829 | non-HS n = 2489 | p value |
| --- | --- | --- | --- | --- |
| Overall | 77.2 ± 17.7 | 83.6 ± 13.9 | 75 ± 18.3 | <0.001 |
| Symptoms | 79.9 ± 19.6 | 86.1 ± 15.1 | 77.9 ± 20.4 | <0.001 |
| Daily activities | 76 ± 22.5 | 84.8 ± 17.0 | 73 ± 23.3 | <0.001 |
| Treatment concerns | 76.7 ± 18.4 | 80.1 ± 16.6 | 75.5 ± 18.9 | <0.001 |
| Satisfaction | 68.4 ± 19.8 | 69.8 ± 19.9 | 68 ± 19.7 | 0.042 |
| 1. Palpitations | 2.7 ± 1.7 | 2.2 ± 1.4 | 2.9 ± 1.8 | <0.001 |
| 2. Irregular heartbeat | 2.5 ± 1.6 | 2.1 ± 1.3 | 2.7 ± 1.7 | <0.001 |
| 3. A pause in heart activity | 1.4 ± 1.0 | 1.3 ± 0.7 | 1.5 ± 1.1 | <0.001 |
| 4. Lightheadedness or dizziness | 2.1 ± 1.4 | 1.8 ± 1.1 | 2.1 ± 1.4 | <0.001 |
| 5. Recreational pastimes, sports, and hobbies | 2.1 ± 1.6 | 1.7 ± 1.2 | 2.3 ± 1.7 | <0.001 |
| 6. Doing things with friends | 1.8 ± 1.3 | 1.4 ± 0.9 | 1.7 ± 1.4 | <0.001 |
| 7. Avoiding activities because of tiredness | 2.3 ± 1.4 | 1.8 ± 1.1 | 2.4 ± 1.5 | <0.001 |
| 8. Avoiding activities owing to shortness of breath | 2.3 ± 1.4 | 1.9 ± 1.1 | 2.5 ± 1.5 | <0.001 |
| 9. Exercising | 2.5 ± 1.6 | 2 ± 1.2 | 2.7 ± 1.7 | <0.001 |
| 10. Walking briskly | 2.4 ± 1.5 | 1.9 ± 1.2 | 2.6 ± 1.6 | <0.001 |
| 11. Walking briskly uphill | 2.9 ± 1.7 | 2.3 ± 1.4 | 3.1 ± 1.8 | <0.001 |
| 12. Doing vigorous activities | 3.2 ± 2.0 | 2.5 ± 1.6 | 3.4 ± 2.0 | <0.001 |
| 13. Feeling worried that AF can start anytime | 2.8 ± 1.6 | 2.3 ± 1.3 | 2.9 ± 1.7 | <0.001 |
| 14. Worry that AF may worsen other conditions | 3.1 ± 1.6 | 2.8 ± 1.5 | 3.2 ± 1.7 | <0.001 |
| 15. Worry about side effects from medications | 2.2 ± 1.3 | 2 ± 1.2 | 2.3 ± 1.4 | <0.001 |
| 16. Worry about side effects from ablation | 2.2 ± 1.5 | 2.2 ± 1.4 | 2.3 ± 1.5 | 0.139 |
| 17. Worry about side effects of blood thinners | 2 ± 1.2 | 1.9 ± 1.1 | 2 ± 1.2 | 0.006 |
| 18. Worry that treatment interferes | 2 ± 1.2 | 2 ± 1.2 | 2.1 ± 1.3 | 0.029 |
| 19. Satisfaction in heart rate control | 2.9 ± 1.2 | 2.8 ± 1.2 | 2.9 ± 1.2 | 0.020 |
| 20. Satisfaction in symptoms | 2.9 ± 1.2 | 2.8 ± 1.2 | 2.9 ± 1.2 | 0.105 |

**QoL score at one year after registration**

|  | All n = 3318 | HS n = 829 | non-HS n = 2489 | p value |
| --- | --- | --- | --- | --- |
| Overall | 84.9 ± 14.5 | 88.2 ± 12.3 | 83.8 ± 15.0 | <0.001 |
| Symptoms | 89.3 ± 13.9 | 92.2 ± 11.7 | 88.3 ± 14.5 | <0.001 |
| Daily activities | 82.6 ± 18.8 | 87.2 ± 15.7 | 81 ± 19.4 | <0.001 |
| Treatment concerns | 84.9 ± 14.2 | 86.4 ± 13.1 | 84.4 ± 14.5 | 0.001 |
| Satisfaction | 65.9 ± 23.6 | 66.1 ± 23.6 | 65.8 ± 23.6 | 0.768 |
| 1. Palpitations | 1.9 ± 1.2 | 1.7 ± 1.0 | 1.9 ± 1.2 | <0.001 |
| 2. Irregular heartbeat | 1.2 ± 0.7 | 1.2 ± 0.6 | 1.2 ± 0.7 | 0.024 |
| 3. A pause in heart activity | 1.7 ± 1.2 | 1.5 ± 1.0 | 1.8 ± 1.2 | <0.001 |
| 4. Lightheadedness or dizziness | 1.7 ± 1.3 | 1.5 ± 1.0 | 1.8 ± 1.4 | <0.001 |
| 5. Recreational pastimes, sports, and hobbies | 1.5 ± 1.0 | 1.3 ± 0.7 | 1.5 ± 1.0 | <0.001 |
| 6. Doing things with friends | 1.8 ± 1.1 | 1.6 ± 1.0 | 1.9 ± 1.1 | <0.001 |
| 7. Avoiding activities because of tiredness | 1.8 ± 1.1 | 1.6 ± 1.0 | 1.9 ± 1.2 | <0.001 |
| 8. Avoiding activities owing to shortness of breath | 2 ± 1.4 | 1.8 ± 1.1 | 2.1 ± 1.4 | <0.001 |
| 9. Exercising | 2 ± 1.3 | 1.7 ± 1.0 | 2.1 ± 1.4 | <0.001 |
| 10. Walking briskly | 2.4 ± 1.6 | 2.1 ± 1.3 | 2.6 ± 1.6 | <0.001 |
| 11. Walking briskly uphill | 2.7 ± 1.8 | 2.2 ± 1.5 | 2.8 ± 1.9 | <0.001 |
| 12. Doing vigorous activities | 2.1 ± 1.2 | 1.9 ± 1.1 | 2.2 ± 1.3 | <0.001 |
| 13. Feeling worried that AF can start anytime | 2.3 ± 1.3 | 2.2 ± 1.2 | 2.3 ± 1.3 | 0.032 |
| 14. Worry that AF may worsen other conditions | 1.8 ± 1.1 | 1.7 ± 1.0 | 1.8 ± 1.1 | 0.037 |
| 15. Worry about side effects from medications | 1.6 ± 1.0 | 1.6 ± 1.0 | 1.6 ± 1.1 | 0.382 |
| 16. Worry about side effects from ablation | 1.8 ± 1.1 | 1.7 ± 1.0 | 1.8 ± 1.1 | 0.051 |
| 17. Worry about side effects of blood thinners | 1.6 ± 0.9 | 1.5 ± 0.9 | 1.6 ± 0.9 | 0.075 |
| 18. Worry that treatment interferes | 2.3 ± 1.1 | 2.1 ± 1.1 | 2.3 ± 1.1 | <0.001 |
| 19. Satisfaction in heart rate control | 2.2 ± 1.2 | 2.1 ± 1.1 | 2.3 ± 1.2 | <0.001 |
| 20. Satisfaction in symptoms | 3.4 ± 1.8 | 3.5 ± 1.7 | 3.4 ± 1.8 | 0.260 |

Values reflect mean ± standard deviation.

AF, atrial fibrillation; HS, health screening; QoL, quality of life

**Supplementary Table 3.** Comparison of QoL scores obtained at baseline and at 1-year follow-up visit

**All patients**

|  | Baseline | Follow-up | p value |
| --- | --- | --- | --- |
| Overall | 77 ± 17.6 | 84.9 ± 14.5 | <0.001 |
| Symptoms | 79.6 ± 19.6 | 89.3 ± 13.9 | <0.001 |
| Daily activities | 76 ± 22.4 | 82.6 ± 18.7 | <0.001 |
| Treatment concerns | 76.4 ± 18.4 | 84.9 ± 14.2 | <0.001 |
| Satisfaction | 68.3 ± 19.8 | 66.3 ± 23.4 | 0.158 |
| 1. Palpitations | 2.8 ± 1.7 | 1.8 ± 1.2 | <0.001 |
| 2. Irregular heartbeat | 2.5 ± 1.6 | 1.2 ± 0.7 | <0.001 |
| 3. A pause in heart activity | 1.4 ± 1.0 | 1.7 ± 1.2 | <0.001 |
| 4. Lightheadedness or dizziness | 2.1 ± 1.4 | 1.7 ± 1.3 | <0.001 |
| 5. Recreational pastimes, sports, and hobbies | 2.1 ± 1.6 | 1.5 ± 1.0 | <0.001 |
| 6. Doing things with friends | 1.8 ± 1.3 | 1.8 ± 1.1 | 0.700 |
| 7. Avoiding activities because of tiredness | 2.3 ± 1.4 | 1.8 ± 1.1 | <0.001 |
| 8. Avoiding activities owing to shortness of breath | 2.3 ± 1.4 | 2 ± 1.4 | <0.001 |
| 9. Exercising | 2.5 ± 1.6 | 2 ± 1.3 | <0.001 |
| 10. Walking briskly | 2.4 ± 1.5 | 2.4 ± 1.6 | 0.353 |
| 11. Walking briskly uphill | 2.9 ± 1.7 | 2.7 ± 1.8 | <0.001 |
| 12. Doing vigorous activities | 3.2 ± 2.0 | 2.1 ± 1.2 | <0.001 |
| 13. Feeling worried that AF can start anytime | 2.8 ± 1.6 | 2.3 ± 1.3 | <0.001 |
| 14. Worry that AF may worsen other conditions | 3.1 ± 1.6 | 1.8 ± 1.1 | <0.001 |
| 15. Worry about side effects from medications | 2.2 ± 1.3 | 1.6 ± 1.0 | <0.001 |
| 16. Worry about side effects from ablation | 2.3 ± 1.5 | 1.8 ± 1.1 | <0.001 |
| 17. Worry about side effects of blood thinners | 2 ± 1.2 | 1.6 ± 0.9 | <0.001 |
| 18. Worry that treatment interferes | 2 ± 1.2 | 2.3 ± 1.1 | <0.001 |
| 19. Satisfaction in heart rate control | 2.9 ± 1.2 | 2.3 ± 1.1 | <0.001 |
| 20. Satisfaction in symptoms | 2.9 ± 1.2 | 3.4 ± 1.8 | <0.001 |

**HS group**

|  | Baseline | Follow-up | p value |
| --- | --- | --- | --- |
| Overall | 83.7 ± 13.6 | 88.2 ± 12.3 | <0.001 |
| Symptoms | 86 ± 14.9 | 92.3 ± 11.7 | <0.001 |
| Daily activities | 84.8 ± 16.9 | 87.2 ± 15.7 | <0.001 |
| Treatment concerns | 80.3 ± 16.1 | 86.5 ± 13.1 | <0.001 |
| Satisfaction | 69.7 ± 20.0 | 66.9 ± 23.0 | 0.11 |
| 1. Palpitations | 2.2 ± 1.3 | 1.7 ± 1.0 | <0.001 |
| 2. Irregular heartbeat | 2.1 ± 1.3 | 1.2 ± 0.6 | <0.001 |
| 3. A pause in heart activity | 1.3 ± 0.7 | 1.5 ± 1.0 | <0.001 |
| 4. Lightheadedness or dizziness | 1.8 ± 1.1 | 1.5 ± 1.0 | <0.001 |
| 5. Recreational pastimes, sports, and hobbies | 1.7 ± 1.2 | 1.3 ± 0.7 | <0.001 |
| 6. Doing things with friends | 1.4 ± 0.9 | 1.6 ± 1.0 | <0.001 |
| 7. Avoiding activities because you felt tired | 1.8 ± 1.0 | 1.6 ± 1.0 | 0.005 |
| 8. Avoiding activities because short of breath | 1.8 ± 1.1 | 1.7 ± 1.1 | 0.033 |
| 9. Exercising | 2 ± 1.2 | 1.7 ± 1.0 | <0.001 |
| 10. Walking briskly | 1.9 ± 1.2 | 2.1 ± 1.4 | <0.001 |
| 11. Walking briskly uphill | 2.3 ± 1.4 | 2.2 ± 1.6 | 0.045 |
| 12. Doing vigorous activities | 2.5 ± 1.6 | 1.9 ± 1.1 | <0.001 |
| 13. Feeling worried that AF can start anytime | 2.2 ± 1.3 | 2.2 ± 1.2 | 0.337 |
| 14. Worry that AF may worsen other conditions | 2.8 ± 1.5 | 1.7 ± 1.0 | <0.001 |
| 15. Worry about side effects from medications | 2 ± 1.2 | 1.6 ± 1.0 | <0.001 |
| 16. Worry about side effects from ablation | 2.1 ± 1.3 | 1.7 ± 1.0 | <0.001 |
| 17. Worry about side effects of blood thinners | 1.8 ± 1.1 | 1.5 ± 0.9 | <0.001 |
| 18. Worry that treatment interferes | 1.9 ± 1.1 | 2.1 ± 1.1 | 0.001 |
| 19. Satisfaction in heart rate control | 2.8 ± 1.2 | 2.1 ± 1.1 | <0.001 |
| 20. Satisfaction in symptoms | 2.8 ± 1.2 | 3.4 ± 1.7 | <0.001 |

**non-HS group**

|  | Baseline | Follow-up | p value |
| --- | --- | --- | --- |
| Overall | 74.8 ± 18.2 | 83.8 ± 15.0 | <0.001 |
| Symptoms | 77.4 ± 20.5 | 88.3 ± 14.5 | <0.001 |
| Daily activities | 73.1 ± 23.3 | 81.1 ± 19.4 | <0.001 |
| Treatment concerns | 75.1 ± 18.9 | 84.4 ± 14.5 | <0.001 |
| Satisfaction | 67.9 ± 19.8 | 66.1 ± 23.5 | 0.479 |
| 1. Palpitations | 3 ± 1.8 | 1.9 ± 1.2 | <0.001 |
| 2. Irregular heartbeat | 2.7 ± 1.7 | 1.2 ± 0.7 | <0.001 |
| 3. A pause in heart activity | 1.5 ± 1.1 | 1.8 ± 1.2 | <0.001 |
| 4. Lightheadedness or dizziness | 2.1 ± 1.4 | 1.8 ± 1.3 | <0.001 |
| 5. Recreational pastimes, sports, and hobbies | 2.3 ± 1.7 | 1.5 ± 1.0 | <0.001 |
| 6. Doing things with friends | 1.9 ± 1.4 | 1.8 ± 1.1 | 0.075 |
| 7. Avoiding activities because you felt tired | 2.4 ± 1.5 | 1.9 ± 1.2 | <0.001 |
| 8. Avoiding activities because short of breath | 2.5 ± 1.5 | 2.1 ± 1.4 | <0.001 |
| 9. Exercising | 2.7 ± 1.7 | 2.1 ± 1.4 | <0.001 |
| 10. Walking briskly | 2.6 ± 1.6 | 2.6 ± 1.6 | 0.660 |
| 11. Walking briskly uphill | 3.1 ± 1.8 | 2.8 ± 1.9 | <0.001 |
| 12. Doing vigorous activities | 3.4 ± 2.0 | 2.2 ± 1.3 | <0.001 |
| 13. Feeling worried that AF can start anytime | 2.9 ± 1.7 | 2.3 ± 1.3 | <0.001 |
| 14. Worry that AF may worsen other conditions | 3.2 ± 1.7 | 1.8 ± 1.1 | <0.001 |
| 15. Worry about side effects from medications | 2.3 ± 1.4 | 1.6 ± 1.1 | <0.001 |
| 16. Worry about side effects from ablation | 2.3 ± 1.5 | 1.8 ± 1.1 | <0.001 |
| 17. Worry about side effects of blood thinners | 2 ± 1.3 | 1.6 ± 0.9 | <0.001 |
| 18. Worry that treatment interferes | 2.1 ± 1.2 | 2.3 ± 1.1 | <0.001 |
| 19. Satisfaction in heart rate control | 3 ± 1.2 | 2.3 ± 1.2 | <0.001 |
| 20. Satisfaction in symptoms | 2.9 ± 1.2 | 3.4 ± 1.8 | <0.001 |

Values reflect mean ± standard deviation.

AF, atrial fibrillation; HS, health screening; QoL, quality of life

**Supplementary Table 4.** Comparison of QoL scores obtained at baseline and at the 1-year follow-up visit in the HS group that did not receive anticoagulants at baseline

**HS group not receiving anticoagulants at baseline**

|  | Baseline | Follow-up | p value |
| --- | --- | --- | --- |
| Overall | 86.6 ± 12.4 | 89 ± 12.8 | 0.015 |
| Symptoms | 87.4 ± 13.3 | 92.4 ± 12.3 | <0.001 |
| Daily activities | 89.8 ± 14.7 | 89 ± 15.3 | 0.484 |
| Treatment concerns | 81.2 ± 15.6 | 86.2 ± 14.2 | <0.001 |
| Satisfaction | 71 ± 20.1 | 67.2 ± 23.8 | 0.187 |
| 1. Palpitations | 2.2 ± 1.2 | 1.7 ± 1.1 | <0.001 |
| 2. Irregular heartbeat | 2 ± 1.1 | 1.2 ± 0.7 | <0.001 |
| 3. A pause in heart activity | 1.3 ± 0.8 | 1.3 ± 0.8 | 0.935 |
| 4. Lightheadedness or dizziness | 1.5 ± 0.8 | 1.5 ± 1.1 | 0.733 |
| 5. Recreational pastimes, sports, and hobbies | 1.4 ± 1.1 | 1.2 ± 0.7 | 0.025 |
| 6. Doing things with friends | 1.2 ± 0.7 | 1.5 ± 0.9 | <0.001 |
| 7. Avoiding activities because of tiredness | 1.5 ± 0.8 | 1.5 ± 0.9 | 0.850 |
| 8. Avoiding activities owing to shortness of breath | 1.6 ± 1 | 1.7 ± 1.2 | 0.626 |
| 9. Exercising | 1.7 ± 1.2 | 1.5 ± 0.9 | 0.033 |
| 10. Walking briskly | 1.6 ± 1 | 1.8 ± 1.3 | 0.003 |
| 11. Walking briskly uphill | 1.9 ± 1.2 | 2 ± 1.5 | 0.308 |
| 12. Doing vigorous activities | 1.9 ± 1.3 | 2 ± 1.2 | 0.894 |
| 13. Feeling worried that AF can start anytime | 2.3 ± 1.3 | 2.3 ± 1.2 | 0.863 |
| 14. Worry that AF may worsen other conditions | 2.9 ± 1.4 | 1.7 ± 1 | <0.001 |
| 15. Worry about side effects from medications | 1.9 ± 1.3 | 1.7 ± 1.1 | 0.142 |
| 16. Worry about side effects from ablation | 2.1 ± 1.3 | 1.6 ± 1 | 0.001 |
| 17. Worry about side effects of blood thinners | 1.5 ± 0.9 | 1.6 ± 1.1 | 0.256 |
| 18. Worry that treatment interferes | 1.9 ± 1.2 | 2.1 ± 1.2 | 0.265 |
| 19. Satisfaction in heart rate control | 2.7 ± 1.3 | 2.1 ± 1.2 | <0.001 |
| 20. Satisfaction in symptoms | 2.7 ± 1.2 | 3.4 ± 1.7 | 0.003 |

Values reflect mean ± standard deviation.

AF, atrial fibrillation; HS, health screening; QoL, quality of life
